# ArrhythmoGenoPharmacoTherapy

**DOI:** 10.3389/fphar.2020.00616

**Published:** 2020-05-12

**Authors:** Arpad Tosaki

**Affiliations:** Department of Pharmacology, School of Pharmacy, University of Debrecen, Debrecen, Hungary

**Keywords:** genetics, ischemia—reperfusion, electrocardiogram (ECG), arrhythmia < cardiovascular, therapy -, action potential (AP)

## Abstract

This review is focusing on the understanding of various factors and components governing and controlling the occurrence of ventricular arrhythmias including (i) the role of various ion channel-related changes in the action potential (AP), (ii) electrocardiograms (ECGs), (iii) some important arrhythmogenic mediators of reperfusion, and pharmacological approaches to their attenuation. The transmembrane potential in myocardial cells is depending on the cellular concentrations of several ions including sodium, calcium, and potassium on both sides of the cell membrane and active or inactive stages of ion channels. The movements of Na^+^, K^+^, and Ca^2+^ *via* cell membranes produce various currents that provoke AP, determining the cardiac cycle and heart function. A specific channel has its own type of gate, and it is opening and closing under specific transmembrane voltage, ionic, or metabolic conditions. APs of sinoatrial (SA) node, atrioventricular (AV) node, and Purkinje cells determine the pacemaker activity (depolarization phase 4) of the heart, leading to the surface manifestation, registration, and evaluation of ECG waves in both animal models and humans. AP and ECG changes are key factors in arrhythmogenesis, and the analysis of these changes serve for the clarification of the mechanisms of antiarrhythmic drugs. The classification of antiarrhythmic drugs may be based on their electrophysiological properties emphasizing the connection between basic electrophysiological activities and antiarrhythmic properties. The review also summarizes some important mechanisms of ventricular arrhythmias in the ischemic/reperfused myocardium and permits an assessment of antiarrhythmic potential of drugs used for pharmacotherapy under experimental and clinical conditions.

## The Heart and Arrhythmias

The heart is the key center of the blood circulation in fish, reptiles, birds, and mammals, an organ contracts autonomously and rhythmically, and functioning in conjunction with an extensive network of blood vessels to supply all cells and organs with oxygen and nutrients for serving their physiological function. Do we really need another review on cardiac arrhythmias in a special issue, someone may ask as you browse through several publications in cardiovascular journals. The answer is yes, and the mission of the current review is to present a short and comprehensive analysis of what is currently known about ischemia- and reperfusion-induced arrhythmias and their mechanisms, focusing on changes in shapes and waves in action potential (AP) and electrocardiogram (ECG), respectively, in animal models and human beings. The human heart produces left ventricular contraction about 70 times in a minute under physiological conditions. The duration and shape of APs are controlled and determined by the function and activity of various ion channels and genes in an individual cardiac cell. In various myocardial cell types, the duration of AP is varied from species to species and the beat-to-beat variability for the AP duration is an important arrhythmogenic predictor, determining the intensity of cell-to-cell coupling (Magyar et al., 2016; Nanasi et al., 2017).

Various techniques for AP recordings and ion current measurements started about 70 years ago and became more sophisticated decade by decade, including the use of monophasic, two-electrode voltage clamp, and whole cell configuration of patch clamp. The recording of resting membrane potential and AP duration have initially been reported by Woodbury et al. (Woodbury et al., 1950), Burgen and Terroux (Burgen and Terroux, 1953), Coraboeuf and Weidmann (Coraboeuf and Weidmann, 1954), and Vaughan Williams (Vaughan Williams, 1959) in cardiac muscle fiber under *in vitro* conditions. Particularly, ion movements *via* voltage-regulated ion channels and pore-regulated proteins across cardiac cell membranes primarily determine the morphology and the duration of the AP in myocardial cells (Sankaranarayanan et al., 2017; Smith and Eisner, 2019). The resting transmembrane potential in an intact cardiac cell is about 85–90 mV negative to the exterior, and the move of Na^+^ into the resting cell leads to the change of the membrane potential and the propagation of the cellular AP. Thus, this movement of Na^+^ determines Ca^2+^ and K^+^ release and exchange mechanisms *via* outer and inner cell membranes and organelles.

Ventricular arrhythmias are the leading cause of sudden cardiac death during and/or following an ischemic episode, acute heart failure, and recovery from myocardial infarction. Life threatening arrhythmias are very common and unpredictable in the failing myocardium; thus, the primary objective of their prevention is to eliminate various environmental risk factors, following the application of appropriate pharmacotherapies or nonpharmacological interventions. Heart failure and sudden ventricular arrhythmia caused deaths are likely to occur before an intervention can be implemented (Ramirez et al., 2014; Ramirez et al., 2017). The tool used for the detection and diagnosis of cardiac arrhythmias is the ECG, thus, the aim of antiarrhythmic therapy could be considered for acute termination or long-term treatment to prevent recurrence of various types of arrhythmias. However, antiarrhythmic drugs have multiple effects on the generation of AP and the waves of ECG, and their mechanisms are very complex (Alvarez et al., 2019; Paar et al., 2019). In addition, an antiarrhythmic agent could modulate other targets and tissues in the different parts of the myocardium in comparison with its primary site of action. In other words, an arrhythmia type and its origin could result from multiple underlying mechanisms at the same time, which may result in either from increased or decreased various types of K^+^, Na^+^, and Ca^2+^ currents *via* cell membranes and cell organelles. Thus, pharmacological therapies of cardiac arrhythmias should be focused on the most relevant underlying mechanism of arrhythmias, where it's known, and interventions may be antiarrhythmic by suppressing the initiating arrhythmogenic mechanism. Atrial fibrillation (AF) is caused by various mechanisms and one of the most common cardiac rhythm disturbances (Chua et al., 2019; Sutanto et al., 2019) associated with increased risk of heart failure, stroke (Kostopoulou et al., 2019), and death. More than dozens of genetic loci have been associated with AF, but the current review does not attempt to focus on these genetic mechanisms (Kalsto et al., 2019) and clinical management (Sutanto et al., 2019) of AF in detail.

## The Action Potential

Ion currents through cell membranes critically determine the shape of the AP and the waves of the ECG, which serve for the recognition and diagnosis of various types of cardiac arrhythmias. Namely, Na^+^ currents, L- and T-type Ca^2+^ currents, transient outward currents, inward rectifier and pacemaker currents, *delayed rectifier K^+^ currents*, Na^+^–Ca^2+^-exchanger (NCX), and Na^+^–K^+^-ATPase pump are the basic components of the AP development. In each AP, Na^+^ entry and K^+^ efflux and exchanges are the initial processes for determining of other ion concentrations and functions inside the cardiac cell. The ATP-depending exchange mechanism, called Na^+^–K^+^-pump, serves to determine and maintain intracellular ion homeostasis, which is electrogenic, and generating a net K^+^ current. Under physiological conditions, intracellular Ca^2+^ is maintained at very low level in cardiac cells, and the entry of Ca^2+^ during AP *via* L-type Ca^2+^ channels is the primary signal to the sarcoplasmic reticulum (SR) to release Ca^2+^ ions from its store, leading to the initiation of Ca^2+^-dependent contraction termed excitation–contraction coupling. Ca^2+^ efflux from SR into the cytoplasm happens *via* ryanodine receptor release channels (RyRc), and the subsequent elimination of intracellular Ca^2+^ from cytoplasm occurs by SERCA, which pumps Ca^2+^ ions back to the SR. In the meantime, the electrogenic NCX located on the cardiac cell membrane surface exchanges three Na^+^ from the exterior for each Ca^2+^ ion extruded. During the past three decades, intensive investigation focused on the ion currents in AP phases, which were connected to specific channel-controlled genes, encoding the major pore-regulated proteins.

## The AP, Ion Channels, and Genes

The identification of specific ion channels and their encoded proteins has contributed to a better understanding of the physiological and pathological APs and their changes, which affect the shape of ECG waves, leading to the mechanisms and diagnosis of various types of arrhythmias and discovery of potential new antiarrhythmic drug targets.

### SCN5A

**Figure 1:**
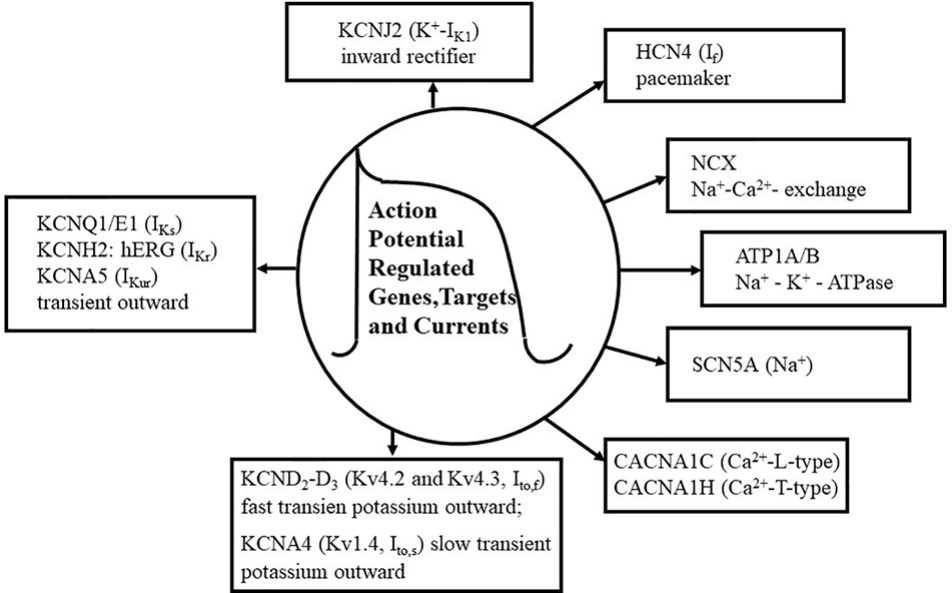
Action potential (AP) genes and ion channels. The figure shows the summary of basic ion currents, genes and their targets in the AP. Various types of Ca^2+^ currents are shown, including L-type and T-type Ca^2+^ currents (CACNA1C, CACNA1H). KCND2-D3 genes (Kv4.2, Kv4.3), as voltage-gated fast transient outward potassium (I_to,f_) and slow transient K^+^ outward (I_to,s_) channels are also shown. Additionally, Na^+^ and K^+^ channels and their pore-regulated proteins and voltage regulations are also depicted. The Na^+^ current (SCN5A) is about 50 times lager as any other current during the depolarization phase (phase 1) of the AP, although its portion persists in the plateau phase. Several types of Ca^2^^+^ currents e.g., CACNA1C (L-type) and CACNA1H (T-type), are activated during the phases 0, 1, and 2 of the AP. Fast transient outward potassium currents (KCND2-D3, encoding Kv4.2-Kv4.3) are functioning in the phases of 0, 1, and 2 of the AP. The activation of KCDN2 is rapidly terminated in the “notch” phase, phase 1, of the AP. Rectifier potassium channels include I_Ks_ (KCNQ1, KCNE1), I_Kr_ (KCNH2, hERG), and I_Kur_ (KCNA5), which are also activated in the phases of 0, 1, and 2 of the AP. Inward rectifier current (KCNJ2), pacemaker current (HCN4, I_f_), and Na^+^–K^+^-ATPase (ATP1A/B) are activated in the phase 4 of the AP. Na^+^–Ca^2+^ exchange mechanism (NCX) is functioning in the phases of 1, 2, and 3 of the AP.

The primary gene (SCN5A) encodes the pore-forming alpha subunit of the primary Na^+^ channel (Na_v1.5_) in the human heart (Lieve et al., 2017; Verkerk et al., 2018), and this so-called “gain-of-function mutation” determines the late Na^+^ current (I_Na_), which leads to (i) the development of one type of long-QT syndrome (LQTS), (ii) the prolongation of the duration of the AP, and (iii) the genesis of premature ventricular contractions in myocardial cells (Keating and Sanguinetti, 2001; Splawski et al., 2002; Wang et al., 2004; Amin et al., 2018). In addition, it was found that genetic mutations of SCN5A variants, including Na_v1.5_, determine I_Na_ and the development of both the LQTS and Brugada syndromes (Tse et al., 2017; Li et al., 2018). Therefore, interventions, which inhibit the mutated gene's (SCN5A) function and/or the abnormal late Na^+^ current (**Figure 1**) may prevent the development of LQTS. In contrast, drugs enhance the late Na^+^ current may cause both atrial and ventricular arrhythmias; called proarrhythmic agents.

### KCND2-D3

Transient outward potassium currents (KCND2-D3, Kv4.2, and Kv4.3) determine the development of the phases (0, 1, and 2) of the AP, however, the activation of KCND2 (Kv4.2) is rapidly terminated in the “notch” phase of the AP. It has been reported that KCND2 and KCND3 (Frank-Hansen et al., 2005), encoding of the voltage-gated potassium channel alpha subunits, Kv4.2 and Kv4.3, conducting the fast transient outward K^+^ current (I_to,f_) in the prolongation of AP duration, which may be associated with the development of LQTS in the myocardium. These authors (Frank-Hansen et al., 2005) concluded that mutations of KCND2 and KCND3 are not a frequent cause of LQTS, however, some variants of these two genes may play a significant role in the development of LQTS phenotype. In another study (Perrin et al., 2014), it was shown that KCND2 gene, as a susceptibility gene, contributes to the sudden cardiac death in an anterior J-wave ECG pattern in patients. The results of the aforementioned publication (Perrin et al., 2014) were the first to implicate KCND2 gene (Kv4.2) as a potential cause of an atypical J-wave pattern related to the sudden cardiac death. Considerable attention has been paid to the understanding of the roles of two functionally distinct K^+^ channels; Kv4.2 and Kv4.3 subunits (Niwa and Nerbonne, 2010), which encode the fast transient outward potassium channels (I_to,f_), while Kv1.4 (KCNA4) encodes the function of the slow potassium channels (I_to,s_). Thus, it could be concluded that several signaling cascade mechanisms and regulatory proteins have been implicated in the control and regulation of I_to,f_ and I_to,s_ proteins/channels, regarding the mechanisms of arrhythmogenesis under physiological and various pathological conditions. Although cardiac I_to,f_ and I_to,s_ channels are differently expressed in various mammalian species and contribute to the heterogeneity of the AP and its waveforms, they have a potential importance in the genesis of cardiac arrhythmias. In addition, it has recently been demonstrated by Zhang et al. (2019a), that jingzhaotoxin-V (JZTX-V), a selective inhibitor of A-type potassium channel, plays a crucial role in the regulation of I_to_ potassium channels, supporting its use as a new potential and novel antiarrhythmic agent.

### KCNQ1/E1 and KCNA5

K^+^ currents so-called delayed rectifier currents, including slow (I_Ks_) and ultrarapid (I_Kur_) channels (Zareba et al., 2003), have been dissociated on the basis how quickly they are activated during the AP. However, cardiac KCNQ1/E1 encodes only slow delayed rectifier channels and does not I_Kr_ (rapid delayed rectifier K^+^ current). These K^+^ rectifier channel activations are increased with the time, whereas Ca^2+^ currents are inactivated, leading to the phase-3 (repolarization phase) of AP (**Figure 1**). It was described (Christophersen et al., 2013) that KCNA5 (I_Kur_) has a high frequency of rare variants related to the development of AF, emphasizing that KCNA5 (**Figure 1**) is probably one of the most predominant genes to produce AF (Colman et al., 2017). KCNA5 also encodes hKv1.5, and this genetic alteration increases the electrical activity of cells, therefore, the controlling of hKv1.5 expression and function may result in a proper physiological electrical activity, suggesting that hKv1.5 may be a potential target for the treatment of AF (Xie et al., 2019). Thus, the selective inhibition of hKv1.5 function could be related to the prolongation of atrial AP without an increase in the duration of ventricular AP in human beings.

### KCNH2

KCNH2, initially described and termed hERG, regulates I_Kr_ (rapid delayed rectifier K^+^ channel) and the blocking of I_Kr_ still remains a major issue in the development of antiarrhythmic drugs (Chen et al., 2000; Mitcheson et al., 2000; Mehta et al., 2018) in connection with the pharmacotherapy of LQTS at genetic levels (Mehta et al., 2018; Yin et al., 2018; Cortez et al., 2019; Kerr et al., 2019). The voltage activated potassium channel (Kv11.1) is encoded by the humane-ether-a-go-go related gene (hERG), which predominantly contributes to the electrical activity of the myocardium (Vandenberg et al., 2012; Vasseur et al., 2019). This channel mediates I_Kr_ current (**Figure 1**) during the repolarization phase of the AP (Orvos et al., 2019) and its malfunction plays a critical role in the development of LQTS (Perissinotti et al., 2018; Ng et al., 2019). In an additional elegant study, Wu et al. (2019) described that KCNQ1 mutation interferes with intracellular hERG transport processes, leading to the development of the phenotype LQTS.

### KCNJ2

It was published in an elegant review (Kubo et al., 2005) that inward rectifier K^+^ channel (K_ir_) subfamilies, based on their amino acid sequence alignments, play important roles in various human diseases, including arrhythmogenesis (**Figure 1**). Therefore, pharmacological aspects of K_ir_ channel mediated functions could be expected as therapeutic tools for the treatment of various cardiac arrhythmias. It was suggested that (Yue et al., 2011; Qi et al., 2015) myofibroblasts also play a critical role in arrhythmogenesis in chronic heart failure by increasing extracellular matrix protein production and having a direct electric interaction with myocytes. However, fibroblasts are not excitable cells electrically, but they are able to express various ion channels including I_K1_, and having resting membrane potential about −40 mV (Kamkin et al., 2003). The study by Qi et al. (2015), demonstrated that KCNJ2 (inward rectifier potassium channel; I_K1_) subunit is present in fibroblasts and cardiomyocytes as well. Thus, drug therapies, which alter the function of KCNJ2 ion channels in connection with Ca^2+^ entry may be an important target for the development of other new antiarrhythmic agents, as inward rectifier potassium channel blockers, in failing myocardium (Szuts et al., 2013).

### HCN4, “Funny” Current

The pacemaker current (I_f_ or I_Kf_) is called “funny” current or pacemaker channel, which is an electric current through the HCN4 and subtype genes regulated proteins in the myocardium (**Figure 1**). The “funny” current is a component of the electrical conduction system producing the natural pacemaker activity of the heart. The I_f_ was first described in Purkinje fibers and atrial cells, and has been extensively studied for several decades (Difrancesco and Ojeda, 1980; Mesirca et al., 2013; Mengesha et al., 2017; Monfredi et al., 2018), however, its function is not completely understood in arrhythmogenesis. HCN4 gene-related arrhythmias, including atrioventricular block and AF, have still an unexplored role in arrhythmogenesis, although HCN4 gene (cyclic nucleotide gated 4 channel) is generally accepted as a genetic marker in myocardial pacemaker tissues (Difrancesco, 2015). Thus, myocardial structural abnormalities could be closely associated with HCN4 mutations, leading to the development of cardiac arrhythmias *via* phosphatidyl-inositol 3,4,5-trisphosphate (PIP3) signal transduction mechanism; regulating the function of funny current, controlling the pacemaker activity and heart rate in the myocardium (Difrancesco, 2019). The term of “funny” refers to a mixed Na^+^ and K^+^ permeability through cell membranes and having a very slow physiological kinetic. In addition, an increase in the activation of I_f_ augments Na^+^ influx in the cell, and paradoxically, in a “reverse mode,” elevates intracellular Ca^2+^ accumulation leading to elevated systolic calcium transients. Consequently, the inhibition of I_f_ reduces intracellular Ca^2+^ rise, thus, alters several processes of ventricular remodeling and structural cardiac function, including apoptosis, cardiomyopathy, and arrhythmias (Yampolsky et al., 2019), which may have an important target in clinical managements of arrhythmogenesis.

### NCX

The pharmacological inhibition of NCX protein expression could be a further target and may be an additional advantage for the therapy of heart failure and cardiac arrhythmias under both experimental and clinical conditions (Sipido et al., 2002; Jost et al., 2013; Schwartz et al., 2013; Bogeholz et al., 2016; Devalla et al., 2016; Kohajda et al., 2016; Javidanpour et al., 2018). During the period of diastole in the myocardium, cytosolic free Ca^2+^ is removed by both the reuptake of SERCA (Ca^2+^-ATPase) and transmembrane extrusion by NCX (Voigt et al., 2012). The basic role of NCX has been recognized in two major types of triggered arrhythmias including delayed afterdepolarization (DAD) and early afterdepolarization (EAD). DAD is developed by Ca^2+^ release from the SR under cellular Ca^2+^ overload. The increased Ca^2+^ overload in the cytosol is removed by NCX, causing additional Na^+^ influx and cell membrane depolarization, which leads to DAD. Thus, interventions, which are able to interfere with the NCX could be an effective antiarrhythmic pharmacotherapy in patients, showing DAD signs on AP and/or on ECG recordings. EAD interrupts AP in the phase of repolarization, and not only various Ca^2+^ channel-related intracellular Ca^2+^ overload but even other ion channels and transporters, particularly Na^+^ and K^+^ channels and exchangers, could also contribute to the development of EAD. Under clinical conditions, EAD triggering is a very common event in human, if the heart rate is reduced and extracellular K^+^ concentration is low. Basically, the duration of AP is longer in Purkinje and endocardial cells than in epicardial tissues, therefore, EAD is more frequently induced in Purkinje and endocardial cells in comparison with epicardial cells. Cardiac arrhythmias showing the sign of EAD could be treated by antiarrhythmic drugs, which shorten the AP duration, e.g., antiarrhythmic agents belong to class IB (Vaughan Williams, 1992).

### ATP1alpha/Beta (Na^+^–K^+^-ATP-ase, Na^+^–K^+^-Pump)

It is of interest to note that calcium channels include the structurally homologous family of voltage-gated ion channels, such as CACNA1C-encoded L-type calcium channel (Ca_v_1.2, LTCC), which transports calcium ions into myocytes, regulating the excitation–contraction coupling process, and leading to the development of LQTS phenotype (Estes et al., 2019; Ye et al., 2019). The results of the aforementioned recent publications reveal that a new population of Ca^2+^ channels represents a new “pathological substrates” for LQTS-related arrhythmias. During each AP the cell gains intracellular Na^+^ and loses intracellular K^+^ content. At the end of each AP, for returning to the physiological membrane potential of −90 mV in the myocyte, the Na^+^–K^+^-ATP-ase serves the energy to extrude three Na^+^ ions and enter two K^+^ ions. This process is electrogenic, generating a net outward Na^+^ current during the phases 3 and 4 of the AP. Several decades ago, it was measured and described, using the whole-cell patch-clamp technique that the aforementioned exchange mechanism is generated by the Na^+^–K^+^-pump, leading to the outward membrane current (I_p_) in a single myocyte (Gadsby et al., 1985). Several groups published that peak I_p_ may be the clearing of excess of Na^+^, which is accumulated close to the inner cardiac cell membrane leaflet, if the Na^+^–K^+^-pump is inhibited (Carmeliet, 1992; Aronsen et al., 2013; Garcia et al., 2016). Thus, a restricted Na^+^ diffusion at a subsarcolemmal area in the cardiomyocyte may be a reasonable therapeutic implication (Han et al., 2010; Lu and Hilgemann, 2017) in the treatment of cardiac arrhythmias.

## The ECG and Inherited Syndromes

**Figure 2:**
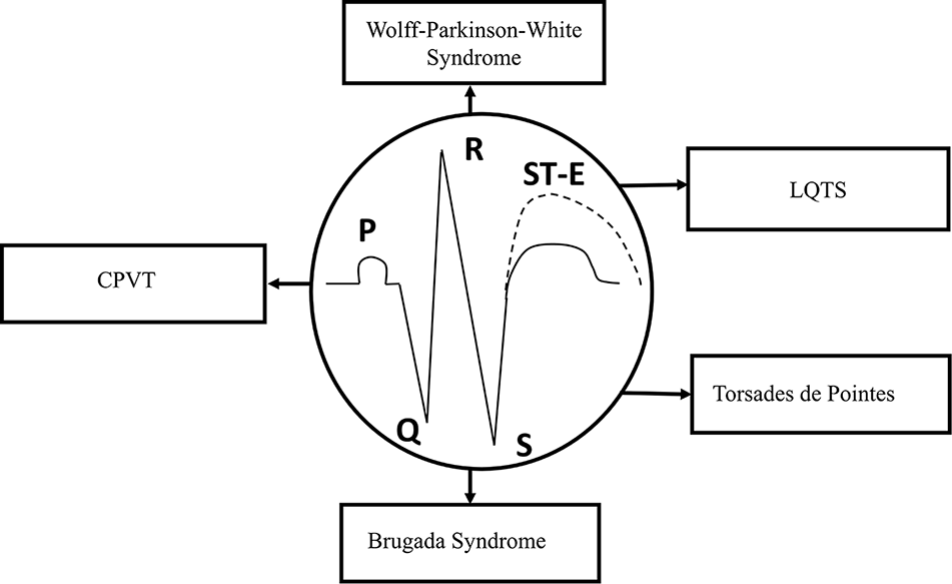
Schematic representation of the ECG. Types of syndromes originated from various gene mutations. Gene mutation-related arrhythmias include long-QT and Brugada syndromes, catecholaminergic polymorphic ventricular tachycardia (CPVT), “torsade de pointes” arrhythmias, and Wolff–Parkinson–White (WPW) syndrome. ST-E, ST-segment elevation.

The duration of APs from different regions of the myocardium, including the sinus node, AV node, and His-Purkinje system, determines the coordinated ventricular contraction, and the electrical activation of the heart, which is normally represented by ECG recordings. In the ECG, P wave, QRS complex, and T wave show the physiological or pathological electrical activity of the myocardium. P wave represents the depolarization phase of the atria, QRS complex indicates the depolarization of the left and right ventricles, while T wave shows the repolarization phase of both ventricles in the ECG (**Figure 2**).

At least, four major inherited syndromes (**Figure 2**) are currently known, which may lead to sudden cardiac death without any structural myocardial diseases, including LQTS, catecholaminergic polymorphic ventricular tachycardia (CPVT), Brugada syndrome, and “torsades de pointes” arrhythmias (Savastano et al., 2014; Delise et al., 2018; Michowitz et al., 2019). However, Wolff–Parkinson–White (WPW) syndrome, which may also be an inherited cardiac disease and leads to reentry ventricular arrhythmias, has some significant structural changes in the myocardial conduction system. Although, these syndromes and sudden consequences were first published several decades ago, the investigation of their genetic origins started about only in the last decade of the twentieth century.

Sodium channels and cardiac structural abnormalities lead to changes in ECG signs and pathological cardiac consequences, which may be associated with SCN5A gene mutation in Brugada syndrome (Coronel et al., 2005). It was suggested that a reduced expression in the number of Na_v_1.5 sodium channels may result in Brugada syndrome in myocardial cells and heterozygous patients (Mohler et al., 2004). Thus, interventions, which could increase the expression of Na_v_1.5 channels in the myocardium may prevent the development of lethal arrhythmias in “Brugada patients,” however, this mechanism should be further investigated.

The increased length of ventricular AP prolongs the QT interval on the ECG, which could be associated with the LQTS and the development of ventricular tachycardia. Four of the aforementioned syndromes (LQT, CPVT, Brugada syndrome, torsades de pointes) appear in patients who did not previously show any cardiac symptoms (Singh et al., 2019) or gross structural changes except syncope before the sudden cardiac death. The ventricular tachycardia with prolonged QT interval is termed “torsades de points” arrhythmia (Bossu et al., 2018). LQT and Brugada syndromes are the most common inherited arrhythmias, while CPVT occurs much rarely.

Dessertenne published (Dessertenne, 1966) initially several original articles about polymorphic ventricular tachycardia in the 1960s, and termed “torsades de pointes” arrhythmia based on ECG signs. Then, it has been the subject of debate for decades, whether “torsades de pointes” is a syndrome or an arrhythmia (Curtis, 1991), and it was concluded that this type of arrhythmia can be a type of arrhythmias and also a syndrome. The final outcomes of the “torsades de pointes” arrhythmia may be syncope, self-terminating, or ventricular fibrillation (VF) leading to sudden cardiac death. The QT interval's prolongation could be genetic origin or acquired (Thomas and Behr, 2016). It was also under extensive investigation (Naksuk et al., 2019) and published that LQTS is a type of arrhythmias, in which “torsades de pointes” arrhythmia causes ventricular tachycardia, VF, and sudden death by the mutation of various genes (KCNQ1/E1 and KCNH2/hERG) underlying potassium repolarization currents, including I_Kr_ and I_Ks_ (Anson et al., 2004; Nerbonne and Kass, 2005; Daubert et al., 2007; Vink et al., 2018; Zhou et al., 2019). In conclusion, clinical management of “torsades de pointes” arrhythmia associated with prolonged QT interval can be pharmacologically treated with intravenous magnesium and potassium infusions for the correction of electrolyte imbalance, and administration of isoproterenol to increase heart rate (Narang and Ozcan, 2019). The application of antiarrhythmic drugs, which prolong the repolarization phase of AP in myocardial cells should be avoided.

The CPVT, which is relatively a rare disease in comparison with the appearance of Brugada and LQTS, applies to an autosomal inherited familial disorder characterized by exercise-induced adrenergic polymorphic ventricular tachycardia, leading to VF and sudden cardiac death (Marks et al., 2002). These “arrhythmogenic” genes are located to the region of genetic locus of chromosome 1q42–q43 as described by Marks et al. (2002), Swan et al. (1999), and Rampazzo et al. (1995). Genetic analyses and findings led to the conclusion that chromosomal area of 1q42–q43 genes is probably closely connected to the function of ryanodine receptor (RyR)–calcium-release channels in cardiac tissues (Priori et al., 2001; Marks et al., 2002). Based on these aforementioned publications, it was suggested that potential inhibitors of RyR, especially RyR2, could be an important antiarrhythmic target for the development of new therapeutic agents (Bongianino et al., 2017; Batiste et al., 2019).

WPW syndrome is defined as the prototype of reentry mechanism, having an accessory connection between the left atrium and left ventricle, and this connection results in a pathological characteristic in QRS complexes. WPW syndrome, based on ECG recordings, was described by these authors in details (Wolff et al., 1930) about ninety years ago. Gollob et al. (2001) first published the gene in familial WPW syndrome, which could be responsible for this type of arrhythmia and sudden cardiac deaths. The authors (Gollob et al., 2001) described a mutation in the encoding gene of gamma subunit of AMP-activated protein kinase in families, which is associated with WPW syndrome, since gamma subunit of AMP-activated protein kinase has a substantial impact in the phosphorylation of several metabolic pathways, including the control of energy substrates in myocardial tissues. Currently, relatively little information is available about the mechanism of AV conduction system and the role of gamma subunit of AMP-activated protein kinase on the control and regulation of various ion channels in familial WPW syndrome (Miyamoto, 2018), although the genetic origin of WPW syndrome and gamma subunit of AMP-activated protein kinase in connection with the reentry mechanism was supported by some recent publications (Bowles et al., 2015; Ben Jehuda et al., 2018). Thus, these findings suggest that gamma subunit of AMP-activated protein kinase and glycogen regulation could be a potent therapeutic target in the treatment of familial WPW syndrome.

### Clinical Management of Prolonged QT interval

Several variations are clinically available for the management of QT prolongation-related arrhythmias (Antoniou et al., 2017). The acceleration of the electrical conduction system in both under experimental and clinical conditions is generally accepted for the management of arrhythmias with prolonged QT interval. Thus, interventions that modify the function of various ion channels, especially Na^+^, K^+^, Ca^2+^, and Mg^2+^ and their exchange mechanisms, could be useful tools for pharmacotherapies of arrhythmias with prolonged QT interval. The QRS wave until the end of the T wave represents the ventricular depolarization and repolarization phases on the ECG. Abnormalities of electrolytes should be corrected with K^+^ infusion monitored continuously, following by intravenous Mg^2+^ sulfate infusion to terminate the prolonged QT interval. In some case, if it is indicated, lidocaine and phenytoin as Na^+^ channel blockers successfully can be also used for the management of LQTS. The beta1/beta2 nonselective adrenergic receptor agonist agent, isoproterenol, shortens QT interval, which could be an effective therapy in humans unresponsive to magnesium sulfate infusion. However, other antiarrhythmic agents initially classified by Vaughan Williams (1975), which are used in clinical therapy against hypoxia- or ischemia-induced arrhythmias e.g., beta adrenergic receptor blockers and calcium channel antagonists, should be avoided for the therapeutic management of arrhythmias with prolonged QT interval. A promising future approach, under experimental conditions to manage the CPVT-initiated QT prolongation, was published by Batiste et al. (2019) showing that an unnatural verticilide enantiomer inhibits the function of RyR2-mediated calcium leak, which inhibits QT prolongation, and prevents life threatening arrhythmias.

The left cardiac sympathetic denervation was also proposed as a useful therapy for LQTS including CPVT (De Ferrari et al., 2015; Desimone et al., 2015; Sgro et al., 2019) in addition to the application of anti‐arrhythmic agents and implantable cardioverter defibrillators. Thus, the combination of these interventions can significantly improve the final outcome of the clinical management of LQTS including CPVT in patients, and the prevention of sudden cardiac death.

## Reperfusion-Induced Injury and Arrhythmias: Major Components

**Figure 3:**
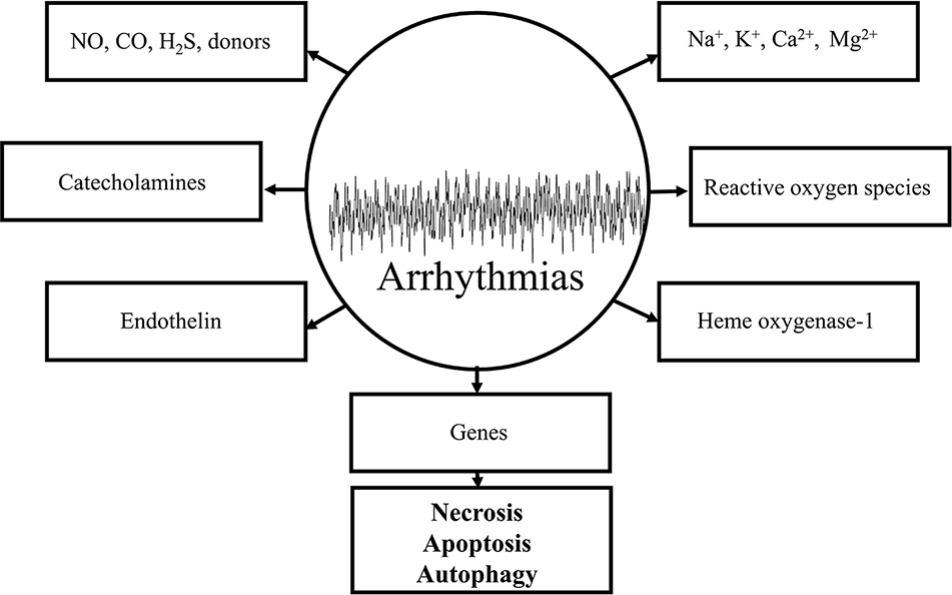
Schematic representation of some important arrhythmogenic components and mediators in the genesis of reperfusion-induced arrhythmias. All of the depicted mechanisms are very complex and each of them significantly contributes to the maldistribution of Na^+^, K^+^, Ca^2+^ exchange mechanisms by virtue of causing damages in cell membranes and receptors leading to necrotic-, apoptotic-, and autophagic-induced cell deaths.

The response of the ischemic myocardium to reperfusion depends on the condition of the tissue at the time it is reperfused. The condition of the myocardium depends on the severity of the ischemia and the duration of the ischemic event. Substantially altered physiological processes in ultrastructural and metabolic changes during ischemia are required for the understanding of the myocardial function to reperfusion. The major ultrastructural and metabolic changes observed in the ischemic myocardium destined to die develop because of the lack of nutrition and oxygen supply and the depressed coronary flow. By definition, the myocardium is considered to be in VF, the most severe life-threatening arrhythmias, if an irregular undulating baseline is observed on the ECG (Walker et al., 1988). The term of “reperfusion-induced” injury caused by VF is originated from the definition itself of the “reperfusion”. Thousands of basic and clinical studies revealed that electrophysiological changes of the ischemic/reperfused myocardium is a dysfunction of cellular homeostasis, which includes the depletion and/or accumulation of various biochemical substances, altering the function of various ion channels and cell-to-cell coupling. Reperfusion-induced arrhythmias include the ectopic beat, tachycardia and fibrillation. The incidence and severity of reperfusion-induced arrhythmias is more frequently occurred under experimental conditions in comparison with human beings (Jennings and Reimer, 1983; Manning and Hearse, 1984; Nakata et al., 1990; Kloner, 1993; Yellon and Hausenloy, 2007; Van Der Weg et al., 2019). Under clinical conditions, reperfusion-induced arrhythmias could mainly be observed during the process of thrombolysis following myocardial ischemia and infarction, after an insult of angina and percutaneous coronary intervention (Corr and Witkowski, 1983; Tzivoni et al., 1983; Tolg et al., 2006; Yu et al., 2017; Van Der Weg et al., 2019). Thrombolytic agents, including urokinase, ataplase, tissue plasminogen activator, are used to dissolve coronary thrombus during early hours of cardiac infarction. The basic arrhythmogenic or proarrhythmogenic role of different mediators, biochemical components, and chemical substances, which accumulate or decompose by virtue of several pathological processes during ischemia/reperfusion in the cellular milieu of the myocardium could be evaluated by various criteria. Therefore, it is important to prove that the antiarrhythmic effect of a drug is primarily originated from its receptor binding activities, and not secondary, e.g., by modifying the coronary blood flow rate. The mission of this chapter is to present a brief summary of what is currently known about the origin of reperfusion-induced arrhythmias, their most important mechanisms, and final outcomes preventing the processes of necrosis, apoptosis and autophagy caused cell deaths under experimental and clinical conditions. The potential importance of major arrhythmogenic components and mediators in reperfusion is shown in **Figure 3**.

The potential mechanisms underlying the genesis of reperfusion-induced arrhythmias have been extensively studied by thousands of investigators. The several pathological processes include the elevation of cAMP (Podzuweit et al., 1978; Oikawa et al., 2013), stimulation of adrenergic receptors (Sheridan et al., 1980; Amirahmadi et al., 2008; Robertson et al., 2014), abnormal lipid metabolism involving lysophosphatides' production (Akita et al., 1986; Axelsen et al., 2015), maldistribution of ionic components through cell membranes, particularly that of Na^+^, Ca^2+^, K^+^, and Mg^2+^, and the genesis of reactive oxygen species (ROS). While a huge variety of pathways may have separately been responsible for the reperfusion-induced arrhythmogenesis, it is reasonable to speculate that all of the aforementioned substances could be directly or indirectly connected to each other, and together they play a crucial final outcome, leading to irreversible arrhythmias, heart failure, and cardiac death.

### K^+^, Na^+^, Ca^2+^, and Mg^2+^

If cardiac tissue is successfully reperfused in the early phase of reversible injury, its reaction is much different from that of the ischemia-induced injury. Myocytes explosively swell, thus, total tissue water content increases about by 20% following the first two min of reperfusion period (Whalen et al., 1974), and tissue electrolyte changes associate with the swelling are striking. As a consequence, there is a significant increase in cellular Na^+^, Ca^2+^, and Cl^−^, while cellular K^+^ and Mg^2+^ are substantially decreased (Whalen et al., 1974).

#### K^+^

Ischemia causes functional disturbance in K^+^ homeostasis in the myocardium and plays a critical role in the genesis of ischemia/reperfusion-induced ventricular arrhythmias (Harris et al., 1958; Tosaki et al., 1988; Curtis and Hearse, 1989). Various mechanisms of drugs by which cellular K^+^ loss causes electrophysiological disturbances, including the function and expression of ATP-dependent K^+^ channels, may involve the blocking and/or opening of these K^+^ channels (Noma, 1983; Tosaki et al., 1995; Das and Sarkar, 2005; Kaya et al., 2019) at various degrees, which may aggravate or diminish the severity of ventricular arrhythmias in the ischemic/reperfused myocardium. Thus, the modification of the expression of K_ATP_ channels by K_ATP_ channel opener or blocker agents, respectively, can increase or decrease the incidence of ischemia/reperfusion-induced arrhythmias. The results of the papers cited above clearly show that interventions, which modify K^+^ transport mechanisms *via* cell membranes, can even increase or reduce the incidence of ischemia/reperfusion-induced arrhythmias.

#### Na^+^

The sodium ion plays a basic process determining the physiological or pathological shape of APs and ECGs in the myocardium. Several mechanisms are currently known, which lead to intracellular Na^+^ accumulation under both physiological and pathological conditions, and the degree of the sodium channel blockade critically determines the membrane potential of the cell and the heart rate. Under pathological conditions, such as myocardial ischemia, the effects of Na^+^/H^+^, Na^+^/K^+^, and Na^+^/Ca^2+^ exchange mechanisms increase the intracellular Ca^2+^ entry, and as a consequence, the ischemic load with additional Ca^2+^ results in a further activation of Na^+^/Ca^2+^ mechanism during reperfusion, leading to the development of reperfusion-induced arrhythmias (Tani and Neely, 1989; Belardinelli et al., 2006). At the early minutes of reperfusion, the substantial accumulation of intracellular Na^+^ during ischemia contributes to a significant increase of cellular edema formation, heart failure, and ventricular arrhythmias. All of these complex mechanisms, without any pharmacological or nonpharmacological interventions, produce an excessive intracellular Ca^2+^ overload leading to necrotic, apoptotic, and autophagic cell deaths. Antiarrhythmic agents, which can modify these pathologic processes, including Na^+^-induced Ca^2+^ overload (Wang et al., 2007), Na^+^/H^+^ exchange mechanism, and Na^+^-induced K^+^ loss could prevent the genesis of reperfusion-induced ventricular arrhythmias and sudden cardiac death. In this respect, the Na^+^/H^+^ exchange mechanism is extensively studied to prevent reperfusion-induced arrhythmias in a variety of experimental models (Karmazyn, 1996; Ravens and Himmel, 1999; Wirth et al., 2001; Woodcock et al., 2001; Ono et al., 2004; Yamada et al., 2005; Szepesi et al., 2015; Hegyi et al., 2018). It can be concluded that Na^+^/H^+^ exchange mechanism, which is not species specific, is a basic target for pharmacological prevention in the attenuation of ischemia/reperfusion-induced cardiac damage, and may emerge as an effective basic therapeutic strategy in human beings. Finally, it is of interest to note that a direct manipulation in extracellular Na^+^ concentration in isolated buffer perfused hearts, a significant decrease in the incidence of reperfusion-induced VF and ventricular tachycardia was detected (Tosaki et al., 1989).

#### Ca^2+^

Under physiological conditions, intracellular Ca^2+^ content is maintained at very low levels, less than 100 nmol. Upon reperfusion, the massive osmotic load in ischemic myocytes, which contain very low level of ATP to fuel the ion channel proteins and pumps results in explosive cell swelling with membrane disruption and myocyte death. Reperfusion-induced arrhythmias are associated with sudden ECG changes, in which there is a Ca^2+^ overload at sufficient level to produce an elevated oscillatory release of Ca^2+^, reflecting in a significant ATP depletion (Coetzee et al., 1987; Coetzee et al., 1988). Thus, the preservation of cell membrane integrity includes maintenance of Ca^2+^ homeostasis, various types of ion exchange receptor-mediated mechanisms, osmotic control, and the preservation of the rich-energy phosphates. Therefore, interventions, e.g., Ca^2+^ channel antagonists (Tosaki et al., 1987a; Otani et al., 2013; Becerra et al., 2016), beta-adrenergic receptor blockers (Tosaki et al., 1987b; Winchester and Pepine, 2014; Gatzke et al., 2018), and several other newly discovered molecular structures and interventions (Wilder et al., 2016; Bukhari et al., 2018; Gatzke et al., 2018), which directly or indirectly contribute to the preservation of the aforementioned processes can diminish the incidence of reperfusion-induced arrhythmias, heart failure, and sudden cardiac death. In addition, during the past three decades several new mechanisms have been proved and implicated based on molecular biological studies, as endogenous mediators, into the pathways of ischemia/reperfusion-induced injury and arrhythmogenesis, including gaseous molecules; nitric monoxide, carbon dioxide, and hydrogen sulfide.

#### Mg^2+^

Magnesium is a “natural” Ca^2+^ channel antagonists (Iseri and French, 1984), a potent cofactor for the function of several enzymes, and plays a crucial role in protein synthesis. Mg^2+^ is also necessary for the insertion of various proteins into cell membranes, and stabilizes the structure of ribosomes (Flatman, 1984). A variety of disorders including diabetes, hypertension, renal tubular disease, hyperthyroidism, steatorrhea, hepatic and cardiac failure may deplete Mg^2+^ stores causing severe ventricular arrhythmias (Iseri and French, 1984; Lazzerini et al., 2018). It was demonstrated about three decades ago by Tzivoni et al. (1988) that “torsade de pointes” ventricular arrhythmias, which were previously resistant to conventional antiarrhythmic therapies, were prevented and controlled by the infusion of magnesium sulfate. In addition, it was also published by other investigators (Tosaki et al., 1993b; Ying et al., 2007; Amoni et al., 2017) that application of Mg^2+^ is a useful tool for the prevention in the development of reperfusion-induced arrhythmias. Although, the precise mechanism of Mg^2+^-induced antiarrhythmic effect is unclear, it has recently been supported that inhibition of the upregulation of P-selectin expression (Ying et al., 2007) and G-protein-coupled receptors (Ye et al., 2018) may be involved in Mg^2+^-induced cardiac protection. The antiarrhythmic effect of Mg^2+^ may be related to the mediation of a number of cellular processes, including particularly the reduction of cellular K^+^ loss, displacement of intracellular Ca^2+^, and maintaining the physiological Mg^2+^ level in myocardial cells. In addition, the possibility that Mg^2+^ could reduce cellular Na^+^ accumulation during ischemia and hence limit intracellular Ca^2+^ overload, could be of considerable importance to its antiarrhythmic effect (Tosaki et al., 1993b).

### Reactive Oxygen Species

Studies focusing on the role of ROS in arrhythmogenesis were first published by Manning and Hearse (Manning and Hearse, 1984) and Woodward and Zakaria (Woodward and Zakaria, 1985). The authors described that ROS, which particularly derive from oxygen, cause ventricular tachycardia and VF during the first minute of the reperfusion period. The arrhythmogenic ROS may arise from several sources in the ischemic/reperfused myocardium including the degradation of catecholamines, the metabolism of arachidonic acid, mitochondrial electron transport processes, and the conversion of xanthine to hypoxanthine by xanthine oxidase. Direct evidence for the burst of ROS generation during the first few minutes of reperfusion has been initially proved by several investigators (Garlick et al., 1987; Zweier and Kuppusamy, 1988; Zweier et al., 1988; Blasig et al., 1990; Tosaki and Braquet, 1990; Samouilov et al., 2019), using electron paramagnetic resonance spectroscopy and its modification. The association between the role of sudden burst of ROS and arrhythmogenesis came also first from electrophysiological studies, proving that ROS generating systems can be potentially arrhythmogenic (Kusama et al., 1989).

The presented results strongly suggest that ROS generation plays a basic role in arrhythmogenesis in reperfusion, and the possibility also exists that ROS formation could interact with other potential pathological mechanisms to facilitate arrhythmogenesis during reperfusion. Therefore, several groups of natural and synthetic pharmacological agents could significantly diminish and/or suppress the incidence of reperfusion-induced arrhythmias. Thus, beta adrenergic receptor blockers (Manning and Hearse, 1984; Lujan et al., 2007; Kloner et al., 2011), calcium channel antagonists (Guc et al., 1993; Podesser et al., 1995; Kato et al., 2004; Bukhari et al., 2018), spin traps (Hearse and Tosaki, 1987; Tosaki et al., 1993a; Zuo et al., 2009; Pisarenko et al., 2019), and natural plants or their extracts (Tosaki et al., 1996; Shen et al., 1998; Broskova et al., 2013; Sedighi et al., 2018; Zhang et al., 2019b) alone, or in their combinations can significantly reduce the severity of ischemia/reperfusion-induced arrhythmias. Several natural products originated from plants, fruits, and/or vegetables showing antiarrhythmic activity by reducing the incidence of reperfusion-induced arrhythmias and preserving myocardial function, include mainly polyphenol and flavonoid molecular structures (Hung et al., 2000; Pataki et al., 2002; Bak et al., 2006; Broskova et al., 2013; Ma et al., 2014; Woodman et al., 2018; Kaya et al., 2019). Natural antioxidant activities and features of flavonoids/polyphenols are important examples of myocardial protection and preservation in ischemic/reperfused hearts, and being not merely a matter of various action mechanisms but showing that differences in molecular formulations may exist among them, even when they are of similar structures.

### Heme Oxygenase-1 and Carbon Monoxide System

Heme oxygenase (HO) catalyzes the first step of heme degradation to carbon monoxide (CO) and biliverdin, and subsequently releases heme iron (Lee et al., 1997; Ryter et al., 1998). CO is an endogenous gaseous molecule and having an important role in hemodynamic regulation in vascular smooth muscle cells (Ishizaka and Griendling, 1997), thus, HO is able to modulate vessel tone, and regulate the changes in blood pressure *via* the increase of cellular cGMP contents by the activation of soluble guanylate cyclase (Johnson et al., 1995).

Two major isozymes of HO exist and produced by two distinct genes (Choi and Alam, 1996); HO-1 is inducible and can be found in various mammalian cells, and HO-2 is constitutively mainly expressed in neurons of the central nervous system. HO-1 expression or repression can be occurred in various disorders (Dulak and Jozkowicz, 2014; Loboda et al., 2016; Haines and Tosaki, 2018) and tissues subjected to oxidative and/or hemodynamic stress (Ottani et al., 2015; Cheng and Rong, 2017). Studies demonstrated that HO-1 expression can afford cellular protection *via* different mechanisms against oxidant stress under both *in vitro* (Lee et al., 1996) and *in vivo* (Nath et al., 1992; Waldman et al., 2019; Yu et al., 2019) conditions. It has been suggested that HO-1-mediated CO production, as a signal transduction molecule, prevents the development of reperfusion-induced VF (Csonka et al., 1999b; Pataki et al., 2001; Bak et al., 2003; Bak et al., 2010). Liang et al. (2014) published that CO prolongs the duration of AP by the inhibition of inward rectifying K^+^ channels. These authors also demonstrated that CO inhibits the function of Kir2.2 and Kir2.3 inward rectifier channels in myocardial cells, and directly affects Kir2.3 function *via* the phosphatidylinositol(4,5)-bisphosphate system. The aforementioned findings show that the inhibition of Kir2.2 and Kir2.3 leads to a substantial prolongation of AP, which may be responsible for the development of reperfusion-induced VF. In addition, Ottani A et al. (Ottani et al., 2015) demonstrated the beneficial effect of NDP-α-MSH, which associated with the overexpression of HO-1 and Bcl-XL, reducing the incidence of arrhythmias and infarct size in the ischemic/reperfused myocardium. It was also shown that very low concentration of CO added directly to the perfusion buffer protects the ischemic/reperfused myocardium *via* cGMP signaling in isolated hearts (Bak et al., 2005). It is reasonable to believe that manipulation of various pathways with pharmacological interventions in the HO-1/CO system plays an important and preventive role in the genesis of the development of reperfusion-induced arrhythmias and myocardial cell death.

### NO, CO, H_2_S (Gaseous Molecules)

#### NO

Nitric oxide (NO) is an endogenous free-radical-type cell signaling molecule with several therapeutic applications. Under physiological conditions, NO is originated from the terminal nitrogen of L-arginine and produced by NO synthase, which has three different isozymes, including endothelial, neural, and inducible encoded by three different genes. NO activates guanylyl cyclase enzyme leading to the increase of cellular cGMP levels by the dephosphorylation of myosin light chains and reducing cytosolic Ca^2+^ concentration, thereby the relaxation of smooth muscles in a board range of organs in mammalians. Thus, NO as a soluble gas molecule is having several pharmacological potentials and therapeutic applications in various diseases including the central nervous and cardiovascular systems, treatment of pulmonary and arterial hypertension, vasospastic angina, and myocardial infarction. NO is able also to inhibit the aggregation of thrombocytes. The signaling and physiological importance of NO was recognized by the awarding of Nobel Prize in Medicine and Physiology to R. Furchgott, L. Ignaro, and F. Murad in 1998.

The role of NO was also intensively studied in ischemia/reperfusion-induced injury in the myocardium during the past two and half decades (Pabla and Curtis, 1995; Liu et al., 1997; Csonka et al., 1999a; Masini et al., 1999; Varga et al., 1999; Fauconnier et al., 2011; Bienvenu et al., 2017; Van Der Weg et al., 2019). The substantial role and investigation of precise mechanisms of NO, both under physiological or pathological conditions, are not a question of debate in cell signaling and myocardial function. A number of studies show that interventions contributed to NO production *via* different mechanisms significantly reduced the reperfusion-induced injury including the incidence of arrhythmias (Shen et al., 1998; Kawahara et al., 2003; Imaizumi et al., 2008; Egom et al., 2011; Kisvari et al., 2015; Enayati et al., 2018; Jones et al., 2018). Various protective NO-mediated signal mechanisms, including the mitoK-(ATP) (Kawahara et al., 2003), the nuclear factor erythroid 2-related factor (NrF2), the extracellular-signaling-regulated-kinase (ERK) (Imaizumi et al., 2008), and Pak1/Akt1 signaling cascade (Egom et al., 2011) have also been reported.

In addition, organic nitrates used for the therapy of myocardial ischemia as NO donors, activate the soluble guanylyl cyclase increasing cGMP and subsequently reducing the intracellular Ca^2+^ level, and prevent the genesis of ischemia and reperfusion-induced arrhythmias. This latest pharmacologically controlled NO-mediated mechanism by organic nitrates is currently probably one of the most important interventions in the clinical management of myocardial ischemia/reperfusion-induced damages. However, it is of also interest to note that other synthetic molecules, e.g., aspirin and molsidomine, which are currently used in clinical medication, and their modified molecular structures (Bertuglia et al., 2004; Szoke et al., 2019) could release NO, leading to cardiac protection in the ischemic/reperfused myocardium.

#### CO

CO is another vasoactive gaseous molecule in close connection with heme oxygenase-1 system discussed in one of the previous chapters, and having substantial toxicological and physiological importance in living subjects (Peers and Steele, 2012). Several tissues including cardiac myocytes express both HO-1 and HO-2 proteins, and HO-1 expression can be particularly increased by several stress factors (Ewing et al., 1994; Wu et al., 2011; Haines and Tosaki, 2018) including myocardial ischemia/reperfusion (Maulik et al., 1996; Lakkisto et al., 2002) as demonstrated at mRNA and protein levels. Interestingly, homozygote (HO-1^−/−^) knockout mouse hearts subjected to ischemia/reperfusion period produced significantly higher cardiac damage and the incidence of VF in comparison with that of heterozygote (HO-1^+/−^) and wild-type mice (HO-1^+/+^) (Bak et al., 2010; Juhasz et al., 2011). Substantial scientific evidence suggests that several of the beneficial and harmful effects of CO arise from its ability, at dose-dependent manner, to modify signal transduction pathways and activities of ion channel proteins. It is apparent from these publications reviewed here that HO-1 and HO-1-mediated endogenous cellular CO levels have cardioprotective effects against myocardial ischemia/reperfusion-induced arrhythmias, and the pharmacological manipulation of HO-1 expression under severely controlled conditions may have clinical application as an antiarrhythmic agent.

#### H_2_S

Hydrogen sulfide was initially considered as a toxic molecule due to its property to inhibit cytochrome-oxidase c in mitochondrial respiration processes. H_2_S, like CO and NO, is endogenously generated gaseous molecule having substantial metabolic and physiological effects in various tissues including neuronal and myocardial cells. In mammalian tissues, three different enzymes encoded by three distinct genes produce H_2_S: cystathionine-gamma-lyase, cystathionine-beta-synthase, and 3-mercaptopiruvate sulfur transferase. In most cells, cystathionine-gamma-lyase and cystathionine-beta-synthase utilize homocysteine and L-cysteine as substrates to liberate pyruvate, ammonium, and H_2_S (Wang, 2002). A number of studies have reported the cardioprotective effect of H_2_S in myocardial ischemia/reperfusion-induced injury *via* different actions, which include ATP-sensitive K^+^-channel opening (Johansen et al., 2006), cGMP generation (Salloum et al., 2012), Akt-mTORC2 (Zhou et al., 2014), and microRNAs (Ren et al., 2019) mediated mechanisms, lead to the significant reduction of apoptotic and necrotic cellular deaths. Consequently, later on, it was also reported that pharmacological interventions serving as H_2_S donors produced cardiac protection against ischemic/reperfused cellular deaths (Sun et al., 2017; Sun et al., 2018). Currently, relatively little is known about H_2_S-mediated cardiac protection and remodeling against reperfusion-induced arrhythmias, however, growing evidence indicates that H_2_S donors could play a role in the reduction of the incidence of reperfusion-induced arrhythmias (Sun et al., 2015; Testai et al., 2016). Thus, H_2_S releasing molecules may particularly be also considered as potential pharmacological option in the prevention of the development of reperfusion-induced arrhythmias, and may have the ability to protect the myocardium against necrosis- and apoptosis-induced cell death.

## Catecholamines

The role of catecholamines in arrhythmogenesis is not a question of debate, however, sometimes, depending on the experimental conditions, it can be controversial, and these endogenous mediators may have different effects in the genesis of ischemia-induced arrhythmias in comparison with reperfusion-induced rhythm disturbances (Opie and Coetzee, 1988; Kurz et al., 1991; Vanoli et al., 1994). The results of several published papers support the fact that stimulation of adrenergic receptors, especially alpha-1 and beta-1, is arrhythmogenic under ischemic/reperfused conditions (Sheridan et al., 1980; Bolli et al., 1984; Thandroyen et al., 1987; Tosaki et al., 1987b; Yasutake and Avkiran, 1995), and the alpha-1 adrenergic receptor stimulation could be due to the elevated transsarcolemmal Ca^2+^ influx and increased release of this ion from the SR (Thandroyen et al., 1987). Furthermore, the pharmacological blockade of alpha-1 adrenergic stimulation could mediate Na^+^/H^+^ exchange mechanism during reperfusion, leading to the reduction of the incidence of reperfusion-induced VF (Yasutake and Avkiran, 1995). Beta adrenergic receptor blockers could also be effective against arrhythmias by virtue of their ability to directly inhibit catecholamine-induced arrhythmogenesis on beta receptors (Ibanez et al., 2012; Winchester and Pepine, 2014). If this is so, the stimulation of beta adrenergic receptors and the concomitant activation of adenyl cyclase enzyme would appear be directly involved in the development of reperfusion-induced dysrhythmias. Therefore, these observations would suggest that stimulation of beta adrenergic receptors could be involved in the genesis of reperfusion-induced arrhythmias and the beta receptor blockade may be pharmacologically expected to be potentially protective.

## Endothelin

Endothelin (ET) is one of numerous endogenous substances generating and accumulating in the ischemic/reperfused heart, or washed out during reperfusion periods, has been implicated as a potential arrhythmogenic factor. Thus, ET could function as an endogenous peptide-structure mediator in arrhythmogenesis, and has a potentially strong vascular action capable of exacerbating myocardial ischemia and heart failure. ET (“big endothelin,” endothelin-1) was first isolated from aortic endothelial cells (Yanagisawa et al., 1988), and is a potent vasoconstrictor agent, which specifically binds to ET-1 receptors, modulating the function of sodium, calcium and potassium ion channels, leading to pathologic changes on the ECG (Curtis et al., 1993; Brunner and Kukovetz, 1996; Wang et al., 2002; Vago et al., 2004; Adlbrecht et al., 2014; Falk et al., 2018). Hundreds of antiarrhythmic mechanisms, synthetic and nonsynthetic pharmacological agents were proposed, with more or less success, to protect directly or indirectly the myocardium against ET-1-related cardiac dysfunction (Isaka et al., 2007; Na et al., 2007; Du et al., 2008; Chan et al., 2016; Lee et al., 2017). The aforementioned approaches of ET-elicited vascular activities lead to multiple theories without precise and very specific discrimination between mechanisms in terms of their importance in reperfusion-induced arrhythmogenesis. However, it is reasonable to suppose that the cardiac protection is probably originated from (i) the inhibition of ET synthesis (Ceylan et al., 2018), (ii) the direct pharmacological blockade of ET receptors (Wainwright et al., 2005; Kaoukis et al., 2013), (iii) downregulation of various microRNAs (Wang et al., 2019), and (iv) their multiple combinations (i–iii).

## Genetic Origin of Reperfusion-Induced Arrhythmias

At the time of writing, there is no substantial scientific evidence available, opposite to ischemia-induced arrhythmias, about the direct genetic origin of reperfusion-induced arrhythmias, since their origin itself is the “reperfusion event,” and such an approach has not been yet intensively investigated based on its genetic-mediated mechanisms (Szendrei et al., 2002; Kovacs et al., 2013; Ravingerova et al., 2013; Bienvenu et al., 2017; Paar et al., 2019). Therefore, it is reasonable to suppose that some gene encoded proteins and channels are already present upon reperfusion in their inactive forms in the myocardium, which may be immediately or quickly activated upon a reperfusion event, leading to the rapid maldistribution of ionic homeostasis, producing other arrhythmogenic components, and the genesis of reperfusion-induced arrhythmias. In majority of experimental studies, drugs and interventions were added prior to the induction of myocardial ischemia, thereby slowing the rate of ischemia-induced damages so that, less degree of cellular injury is present at the onset of reperfusion in the myocardium in drug-treated groups. Therefore, about a drug-afforded protection or a rapid activation of an inactive gene at the moment of reperfusion can only be obtained from observations, in which drug-induced ion channel activities or gene expression-related changes are immediately studied (or even later) at the onset of reperfusion. Therefore, it is important to consider all the possible inactive/active stages of various genes and function of ion channels at the onset of reperfusion, by which a drug induced cardiac protection might be successfully achieved.

## Conclusion

Myocardial ischemia/reperfusion-induced alterations resulting in heart failure, conduction abnormalities and arrhythmias, in view of structural and electrical remodeling, which are important processes in the development various myocardial diseases. Multiple mechanisms responsible for the development of reperfusion-induced VF, which lead to the sudden cardiac death without pharmacological or nonpharmacological intervention in animal models and post-myocardial infarcted patients.

Potential mechanisms depicted in **Figure 3** show that reperfusion of the ischemic myocardium intensifies the incidence of reperfusion-induced arrhythmias, which are followed by necrosis-, apoptosis-, and autophagy-induced cell deaths (Osipov et al., 2009; Zhang et al., 2012; Chang et al., 2013; Czegledi et al., 2019; Gyongyosi et al., 2019; Yasuda et al., 2019). From a different point of view, autophagic processes may be even beneficial, depending on their intensity in reversible injured myocardial cells. However, necrosis-induced cell death could dominate and mask autophagic signal transduction processes and autophagy-induced cell death in myocardial tissues (Czegledi et al., 2019; Gyongyosi et al., 2019). Cellular biology and genetics have a real value and significant impact to try better to define the generation of cardiovascular diseases, including life threatening cardiac arrhythmias. Nowadays, the research direction and available modern techniques in cell biology are intensively productive, but should do much to clarify gaps between the basic molecular science and clinical relevance in arrhythmogenesis.

## Limitation

Cardiac muscle contraction and relaxation are very complex and primarily determined by several intracellular processes that control the force activation and inactivation, which include Na^+^, K^+^, and Ca^2+^ exchange mechanisms, including the ATP-dependent rate and Ca^2+^ sequestration capacity of the SR. The immediate extrapolation of AP and ECG changes obtained under experimental conditions to an actual clinical situation must be viewed with some caution because of the presence or absence of the blood and its elements (e.g., leukocytes, platelets), signal transductions, and possible interspecies differences in cardiac metabolism. In addition, the duration and shape of APs are different in His-Purkinje cells, AV node, and ventricular tissues, and the refractoriness is basically determined by the voltage-dependent recovery of Na^+^ channels from inactivation returning to activation in close connection with the function of calcium channels. Thus, the duration of effective refractory period is varied in different cells of the heart and the longest interval at which a premature stimuli fails to generate a propagated response, frequently is used to estimate drug effects in physiologically functioning cardiac tissues. Under physiological and pathological experimental conditions, to investigate the effect of an antiarrhythmic agent, the key aim is related to study various changes in the duration of the effective refractory period, which could modify the activation/inactivation stage of Na^+^ channels in close connection with different types of Ca^2+^ channels and genes e.g., Na^+^-induced Ca^2+^ release from the SR. Despite the limitation of several antiarrhythmic drugs can possess protective effects against LQTS and reperfusion-induced arrhythmias even some of them may be of particular concern. Additional experimental and clinical prospective studies powered to determine differences in ventricular APs in connection with ion channels and ECGs to confirm these observations are now warranted.

## Author Contributions

The author confirms being the sole contributor of this work and has approved it for publication.

## Funding

This work was supported by grants of NKFIH-K-124719 and the European Union and the State of Hungary, co-financed by the European Social Fund in the framework of GINOP-2.3.2-15-2016-00043.

## Conflict of Interest

The author declares that the research was conducted in the absence of any commercial or financial relationships that could be construed as a potential conflict of interest.
